# A second polymorph of (*Z*)-3-amino-4-(2-phenyl­hydrazinyl­idene)-1*H*-pyrazol-5(4*H*)-one

**DOI:** 10.1107/S1600536814000427

**Published:** 2014-01-15

**Authors:** Abdel-Sattar S. Hamad Elgazwy, Peter G. Jones

**Affiliations:** a Chemistry Department, Faculty of Science, Ain Shams University, Abbassia 11566, Cairo, Egypt; b Institut für Anorganische und Analytische Chemie, Technische Universität Braunschweig, Postfach 3329, 38023 Braunschweig, Germany

## Abstract

The mol­ecule of the title compound, C_9_H_9_N_5_O, is approximately planar (the r.m.s. deviation of all non-H atoms is 0.08 Å). The amine substituent is pyramidal at the N atom. An intra­molecular N—H_hydrazine_⋯O=C hydrogen bond is present. In the crystal, mol­ecules are connected *via* N—H⋯N and N—H⋯O hydrogen bonds, forming infinite layers parallel to (010). This polymorph is triclinic, space group *P*-1, whereas the previously reported form was monoclinic, space group *P*2_1_/*c* [Elgemeie *et al.* (2013 ▶). *Acta Cryst*. E**69**, o187], with stepped layers and a significantly lower density.

## Related literature   

Synthetic purine (Hamad & Derbala, 2001 ▶) and pyrazole (Elgazwy, 2003 ▶; Madkour & Elgazwy, 2007 ▶) analogues find numerous applications in clinical medicine and medical research. For the synthesis, chemistry, medicinal chemistry and biological activity of related compounds, see: Elgazwy *et al.* (2012*a* ▶,*b* ▶, 2013 ▶); Arnost *et al.* (2010 ▶). For the monoclinic polymorph of the title compound, see: Elgemeie *et al.* (2013 ▶, 2014 ▶). For a description of the Cambridge Structural Database, see: Allen (2002 ▶).
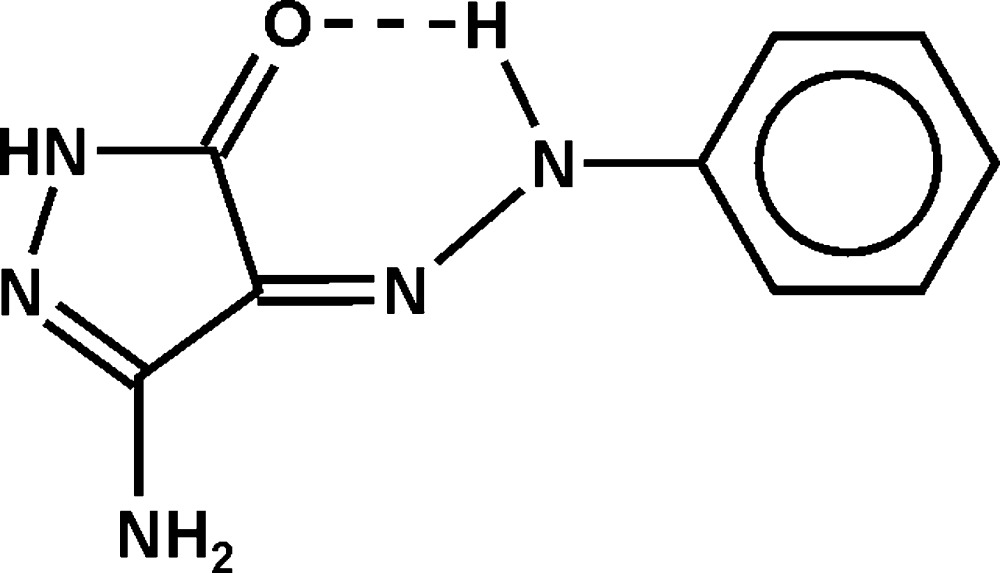


## Experimental   

### 

#### Crystal data  

C_9_H_9_N_5_O
*M* *_r_* = 203.21
Triclinic, 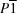

*a* = 6.4433 (4) Å
*b* = 7.4563 (5) Å
*c* = 10.1989 (6) Å
α = 80.005 (5)°
β = 81.271 (5)°
γ = 70.512 (5)°
*V* = 452.57 (5) Å^3^
*Z* = 2
Mo *K*α radiation
μ = 0.11 mm^−1^
*T* = 100 K
0.40 × 0.35 × 0.15 mm

#### Data collection  

Oxford Diffraction Xcalibur Eos diffractometer
Absorption correction: multi-scan (*CrysAlis PRO*; Agilent, 2010 ▶) *T*_min_ = 0.948, *T*_max_ = 1.000
31042 measured reflections
2848 independent reflections
2649 reflections with *I* > 2σ(*I*)
*R*_int_ = 0.022

#### Refinement  

*R*[*F* ^2^ > 2σ(*F* ^2^)] = 0.037
*wR*(*F* ^2^) = 0.107
*S* = 1.04
2848 reflections
152 parameters
H atoms treated by a mixture of independent and constrained refinement
Δρ_max_ = 0.54 e Å^−3^
Δρ_min_ = −0.19 e Å^−3^

### 

Data collection: *CrysAlis PRO* (Agilent, 2010 ▶); cell refinement: *CrysAlis PRO*; data reduction: *CrysAlis PRO*; program(s) used to solve structure: *SHELXS97* (Sheldrick, 2008 ▶); program(s) used to refine structure: *SHELXL97* (Sheldrick, 2008 ▶); molecular graphics: *XP* in *SHELXTL* (Sheldrick, 2008 ▶); software used to prepare material for publication: *SHELXL97*.

## Supplementary Material

Crystal structure: contains datablock(s) I, global. DOI: 10.1107/S1600536814000427/zq2216sup1.cif

Structure factors: contains datablock(s) I. DOI: 10.1107/S1600536814000427/zq2216Isup2.hkl

Supporting information file. DOI: 10.1107/S1600536814000427/zq2216Isup3.cml

CCDC reference:

Additional supporting information:  crystallographic information; 3D view; checkCIF report

## Figures and Tables

**Table 1:** Hydrogen-bond geometry (Å, °)

*D*—H⋯*A*	*D*—H	H⋯*A*	*D*⋯*A*	*D*—H⋯*A*
N5—H05⋯O1	0.920 (14)	2.164 (14)	2.8524 (9)	130.9 (11)
N5—H05⋯O1^i^	0.920 (14)	2.266 (14)	3.0176 (10)	138.6 (12)
N1—H01⋯N2^ii^	0.931 (14)	2.089 (14)	2.9272 (10)	149.0 (12)
N1—H01⋯N1^ii^	0.931 (14)	2.547 (14)	3.0692 (14)	115.8 (10)
N3—H031⋯N2^iii^	0.899 (16)	2.369 (16)	3.2424 (10)	163.9 (13)
N3—H032⋯O1^iv^	0.893 (15)	2.185 (15)	3.0428 (10)	161.0 (13)

Symmetry codes: (i) 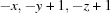; (ii) 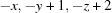; (iii) 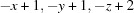; (iv) 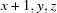.

**Fig. 1.**
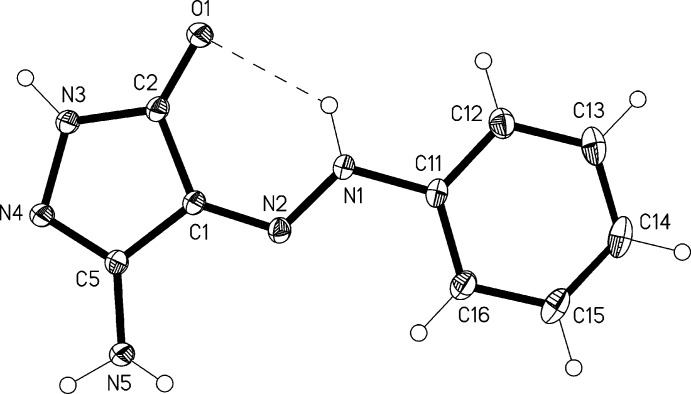
The molecule of the title compound in the crystal; ellipsoids represent 50% probability levels.

**Fig. 2.**
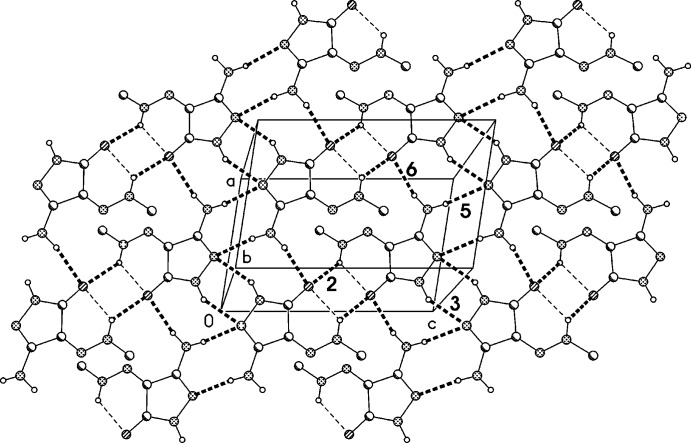
Packing diagram of the title compound viewed perpendicular to the ac plane. Phenyl rings are reduced to the ipso carbons for clarity. Intramolecular hydrogen bonds are drawn as thin and intermolecular hydrogen bonds as thick dashed lines; the latter are numbered according to their order in the relevant Table. H bonds #4 are omitted because they represent the weak components of asymmetric three-centre interactions.

## supplementary crystallographic information

### 1. Comment

Synthetic purine (Hamad & Derbala, 2001) and pyrazole (Elgazwy, 2003; Madkour & Elgazwy, 2007) analogues find numerous applications in clinical medicine and medical research. As part of our work directed towards the synthesis of pyrazolones (Elgazwy *et al.*, 2012*a*, 2012*b*), we have recently reported various successful approaches to the syntheses of pyrazolone analogues that are interesting for biochemical reactions. The title compound (*Z*)-3-amino-4-(2-phenylhydrazono)-1*H*-pyrazol-5(4*H*)-one (I) was prepared in the course of these investigations.

By chance, compound I was also prepared and structurally investigated during a collaboration with a different group (Elgemeie *et al.*, 2013, 2014; regrettably, the stereochemistry was erroneously described as *E*). The current structure proved to be a different polymorph of I, being triclinic (space group *P*1; henceforth I*t*) rather than monoclinic (space group *P*2_1_/*c*; henceforth I*m*).

The molecule of I*t* is shown in Fig. 1. Molecular dimensions, such as the bond lengths C4═N4 1.3070 (10) and N4—N5 1.3128 (9) Å, may be regarded as normal. A search of the Cambridge Database (Allen, 2002; Version 1.15) for the same five-membered ring with C═O and C═N—N substituents gave average bond lengths of 1.309 (8) and 1.318 (10) Å respectively for these bonds (35 hits, 39 fragments excluding one obvious outlier). The nitrogen N3 of the amine substituent is pyramidal, lying 0.224 (8) Å out of the plane of its substituents. The entire molecule is planar to within an r.m.s. deviation of 0.08 Å for the non-H atoms, with no torsion angle deviating by more than 6.5 ° from 0/180 °; this is in part attributable to the intramolecular hydrogen bond N5—H05···O1. A more detailed analysis shows two planar units (i) C11–16 with substituent N5 and (ii) the five-membered ring, both with r.m.s.d. 0.004 Å, that subtend an interplanar angle of 8.51 (4) °. The substituents O1, N4 and N3 all lie slightly but significantly out of the plane of the five-membered ring, by 0.057 (1), 0.095 (1) and 0.069 (1) Å respectively, all to the same side. A comparison with I*m* shows that the molecules are closely similar except for a slight difference in the orientation of the phenyl ring; a least-squares fit excluding the non-*ipso* phenyl carbon atoms gives an r.m.s. deviation of 0.04 Å. However, there are some significant differences such as the N1—C5 bond length, which is 1.3387 (13) Å in I*m* but 1.3489 (10) Å in I*t*.

The molecules of I*t* are connected by hydrogen bonds (Table 1) to form infinite layers (one per b translation) parallel to the *ac* plane (Fig. 2). There are two three-centre interactions (N5—H05···O1 intra- and intermolecular, N1—H01···N1/N2) and two two-centre interactions involving the NH_2_ H atoms (with N2 and O1 as acceptors). In I*m*, one of the N3 H atoms forms a three-centre system with N1 and N2, the N5—H05···O1 interaction is purely two-centre, N1—H01 and N3—H03A are two-centre donors to O1. The packing of I*m* is a stepped layer structure that intuitively seems to display a less efficient packing than that of I*t*; consistent with this are the crystallographic densities of 1.464 and 1.491 g cm^-3^, respectively, but we have carried out no further calculations or experiments to test this hypothesis.

### 2. Experimental

The title compound was obtained according to the following general procedure (Arnost *et al.*, 2010): 3-amino-1*H*-pyrazol-5(4*H*)-one was prepared by refluxing an ethanolic solution of sodium cyanoacetate and sodium ethoxide containing a few drops of hydrazine hydrate for 1 h. After cooling, the precipitate was filtered off and recrystallized from ethanol. A solution of freshly prepared phenyldiazonium chloride (2.66 mmol) was added to the pyrazolone (1 equiv in 50% aqueous EtOH, 3 ml/mmol)and potassium acetate (6 equiv) and the mixture stirred at 0 °C for a further 30 min. The precipitate was filtered, washed with water and dried to give (*Z*)-3-amino-4-(2-phenylhydrazono)-1*H*-pyrazol-5(4*H*)-one (I). Recrystallization from ethanol afforded reddish-brown crystals in 87% yield; m.p. 119–120 °C (dec). Red single crystals of I were obtained by slow diffusion of water into a ethanol solution. Most crystals were swallowtail twins; single crystalline fragments could be separated using a razor blade. IR (KBr, ν (cm^-1^): 3449, 3350, 3330, 3300 (NH_2_, 2NH), 1702, 1668 (C═O), 1627 (C═N), 1475 (N═N); ^1^H NMR (300 MHz, DMSO-d6): δ 6.85 (s, br, 2H, NH_2_), 7.41–7.92 (m, 5H, Ph), 10.71 (s, br, 1H, NH, pyrazole), 14.18 (br, 1H, NH, hydrazone); ^13^C-NMR (DMSO-d6): δ 175.9 (C═O), 162.2 (C-5), 154.3 (C-3), 154.2 (C-4), 143.2 (C-*i*), 129.7 (C-*m*), 120.0 (C-*p*), 117.9 (C-*o*). Anal. Calcd for for C_9_H_9_N_5_O (203); C, 53.20; H, 4.46; N, 34.47. Found: C, 53.27; H, 4.51; N, 34.12%.

### 3. Refinement

The NH H atoms were refined freely. Other H were placed in calculated positions and refined using a riding model with C—H_arom_ 0.95 Å; the hydrogen *U* values were fixed at 1.2 × *U*(eq) of the parent atom for these H.

### Crystal data

**Table:**

C_9_H_9_N_5_O	*Z* = 2
*M**_r_* = 203.21	*F*(000) = 212
Triclinic, *P*1	*D*_x_ = 1.491 Mg m^−^^3^
Hall symbol: -P 1	Melting point = 120–119 K
*a* = 6.4433 (4) Å	Mo *K*α radiation, λ = 0.71073 Å
*b* = 7.4563 (5) Å	Cell parameters from 18469 reflections
*c* = 10.1989 (6) Å	θ = 2.9–31.4°
α = 80.005 (5)°	µ = 0.11 mm^−^^1^
β = 81.271 (5)°	*T* = 100 K
γ = 70.512 (5)°	Tablet, brown-orange dichroic
*V* = 452.57 (5) Å^3^	0.40 × 0.35 × 0.15 mm

### Data collection

**Table:**

Oxford Diffraction Xcalibur Eos diffractometer	2848 independent reflections
Radiation source: Enhance (Mo) X-ray Source	2649 reflections with *I* > 2σ(*I*)
Graphite monochromator	*R*_int_ = 0.022
Detector resolution: 16.1419 pixels mm^-1^	θ_max_ = 31.5°, θ_min_ = 2.9°
ω–scan	*h* = −9→9
Absorption correction: multi-scan (*CrysAlis PRO*; Agilent, 2010)	*k* = −10→10
*T*_min_ = 0.948, *T*_max_ = 1.000	*l* = −14→14
31042 measured reflections	

### Refinement

**Table:**

Refinement on *F*^2^	Primary atom site location: structure-invariant direct methods
Least-squares matrix: full	Secondary atom site location: difference Fourier map
*R*[*F*^2^ > 2σ(*F*^2^)] = 0.037	Hydrogen site location: inferred from neighbouring sites
*wR*(*F*^2^) = 0.107	H atoms treated by a mixture of independent and constrained refinement
*S* = 1.04	*w* = 1/[σ^2^(*F*_o_^2^) + (0.0618*P*)^2^ + 0.1329*P*] where *P* = (*F*_o_^2^ + 2*F*_c_^2^)/3
2848 reflections	(Δ/σ)_max_ < 0.001
152 parameters	Δρ_max_ = 0.54 e Å^−^^3^
0 restraints	Δρ_min_ = −0.19 e Å^−^^3^

### Special details

**Table:**

Geometry. All e.s.d.'s (except the e.s.d. in the dihedral angle between two l.s. planes) are estimated using the full covariance matrix. The cell e.s.d.'s are taken into account individually in the estimation of e.s.d.'s in distances, angles and torsion angles; correlations between e.s.d.'s in cell parameters are only used when they are defined by crystal symmetry. An approximate (isotropic) treatment of cell e.s.d.'s is used for estimating e.s.d.'s involving l.s. planes.Least-squares planes (*x*,*y*,*z* in crystal coordinates) and deviations from them (* indicates atom used to define plane)1.7900 (0.0023) *x* + 6.5551 (0.0012) *y* - 3.2198 (0.0028) *z* = 1.0389 (0.0022)* -0.0005 (0.0005) N5 * 0.0026 (0.0007) C11 * -0.0059 (0.0007) C12 * 0.0039 (0.0006) C13 * 0.0020 (0.0007) C14 * -0.0064 (0.0006) C15 * 0.0043 (0.0007) C16Rms deviation of fitted atoms = 0.00411.7459 (0.0026) *x* + 6.9955 (0.0012) *y* - 1.8138 (0.0040) *z* = 1.9727 (0.0038)Angle to previous plane (with approximate e.s.d.) = 8.51 (0.04)* 0.0048 (0.0005) C4 * -0.0057 (0.0005) C5 * 0.0045 (0.0005) N1 * -0.0011 (0.0005) N2 * -0.0025 (0.0005) C3 - 0.0571 (0.0012) O1 - 0.0953 (0.0013) N4 - 0.0691 (0.0013) N3Rms deviation of fitted atoms = 0.00410.9251 (0.0393) *x* + 7.1132 (0.0235) *y* - 0.8552 (0.1393) *z* = 2.4580 (0.1235)Angle to previous plane (with approximate e.s.d.) = 10.20 (0.77)* 0.0000 (0.0001) C3 * 0.0000 (0.0001) H031 * 0.0000 (0.0000) H032 - 0.2236 (0.0081) N3Rms deviation of fitted atoms = 0.0000
Refinement. Refinement of *F*^2^ against ALL reflections. The weighted *R*-factor *wR* and goodness of fit *S* are based on *F*^2^, conventional *R*-factors *R* are based on *F*, with *F* set to zero for negative *F*^2^. The threshold expression of *F*^2^ > σ(*F*^2^) is used only for calculating *R*-factors(gt) *etc*. and is not relevant to the choice of reflections for refinement. *R*-factors based on *F*^2^ are statistically about twice as large as those based on *F*, and *R*- factors based on ALL data will be even larger.

### Fractional atomic coordinates and isotropic or equivalent isotropic displacement parameters (Å^2^)

**Table:**

	*x*	*y*	*z*	*U*_iso_*/*U*_eq_	
N1	0.07044 (12)	0.48559 (11)	0.85055 (7)	0.01658 (16)	
H01	−0.069 (2)	0.523 (2)	0.8985 (14)	0.026 (3)*	
N2	0.26071 (11)	0.45453 (11)	0.91700 (7)	0.01619 (15)	
C3	0.43103 (13)	0.38930 (11)	0.83013 (8)	0.01377 (16)	
C4	0.36028 (13)	0.37553 (11)	0.70490 (8)	0.01289 (15)	
C5	0.11746 (13)	0.43979 (11)	0.72476 (8)	0.01381 (16)	
C11	0.53773 (13)	0.20424 (11)	0.39128 (8)	0.01417 (16)	
C12	0.43303 (15)	0.18095 (12)	0.28826 (9)	0.01816 (17)	
H12	0.2761	0.2239	0.2922	0.022*	
C13	0.56037 (17)	0.09431 (13)	0.17965 (9)	0.02246 (19)	
H13	0.4900	0.0798	0.1085	0.027*	
C14	0.78986 (17)	0.02890 (13)	0.17463 (9)	0.0239 (2)	
H14	0.8762	−0.0305	0.1004	0.029*	
C15	0.89267 (16)	0.05055 (13)	0.27848 (10)	0.02299 (19)	
H15	1.0495	0.0042	0.2754	0.028*	
C16	0.76831 (14)	0.13954 (12)	0.38721 (9)	0.01822 (17)	
H16	0.8392	0.1559	0.4574	0.022*	
N3	0.64559 (12)	0.33318 (11)	0.85693 (7)	0.01741 (16)	
H031	0.671 (3)	0.370 (2)	0.9308 (16)	0.035 (4)*	
H032	0.743 (2)	0.343 (2)	0.7861 (15)	0.032 (4)*	
N4	0.49280 (11)	0.30196 (10)	0.60388 (7)	0.01314 (14)	
N5	0.40314 (11)	0.29333 (10)	0.49879 (7)	0.01411 (15)	
H05	0.253 (2)	0.345 (2)	0.4940 (14)	0.027 (3)*	
O1	−0.01526 (10)	0.44520 (9)	0.64628 (6)	0.01777 (14)	

### Atomic displacement parameters (Å^2^)

**Table:**

	*U*^11^	*U*^22^	*U*^33^	*U*^12^	*U*^13^	*U*^23^
N1	0.0113 (3)	0.0266 (4)	0.0117 (3)	−0.0051 (3)	0.0009 (2)	−0.0060 (3)
N2	0.0118 (3)	0.0242 (3)	0.0130 (3)	−0.0053 (3)	−0.0002 (2)	−0.0054 (3)
C3	0.0129 (3)	0.0170 (3)	0.0117 (3)	−0.0054 (3)	0.0003 (3)	−0.0029 (3)
C4	0.0116 (3)	0.0157 (3)	0.0113 (3)	−0.0046 (3)	0.0007 (3)	−0.0024 (3)
C5	0.0121 (3)	0.0168 (3)	0.0117 (3)	−0.0042 (3)	0.0011 (3)	−0.0024 (3)
C11	0.0160 (3)	0.0135 (3)	0.0116 (3)	−0.0037 (3)	0.0017 (3)	−0.0027 (3)
C12	0.0208 (4)	0.0175 (4)	0.0155 (4)	−0.0041 (3)	−0.0021 (3)	−0.0041 (3)
C13	0.0330 (5)	0.0195 (4)	0.0143 (4)	−0.0069 (3)	−0.0007 (3)	−0.0051 (3)
C14	0.0314 (5)	0.0192 (4)	0.0179 (4)	−0.0064 (3)	0.0084 (3)	−0.0069 (3)
C15	0.0204 (4)	0.0210 (4)	0.0243 (4)	−0.0045 (3)	0.0078 (3)	−0.0073 (3)
C16	0.0157 (4)	0.0194 (4)	0.0181 (4)	−0.0039 (3)	0.0021 (3)	−0.0050 (3)
N3	0.0124 (3)	0.0268 (4)	0.0137 (3)	−0.0062 (3)	−0.0001 (2)	−0.0057 (3)
N4	0.0139 (3)	0.0142 (3)	0.0113 (3)	−0.0050 (2)	0.0003 (2)	−0.0020 (2)
N5	0.0124 (3)	0.0177 (3)	0.0116 (3)	−0.0034 (2)	0.0005 (2)	−0.0044 (2)
O1	0.0138 (3)	0.0252 (3)	0.0137 (3)	−0.0046 (2)	−0.0017 (2)	−0.0037 (2)

### Geometric parameters (Å, º)

**Table:**

N1—C5	1.3489 (10)	C12—H12	0.9500
N1—N2	1.4227 (10)	C13—C14	1.3898 (15)
N1—H01	0.931 (14)	C13—H13	0.9500
N2—C3	1.3130 (10)	C14—C15	1.3894 (14)
C3—N3	1.3602 (10)	C14—H14	0.9500
C3—C4	1.4502 (11)	C15—C16	1.3939 (12)
C4—N4	1.3070 (10)	C15—H15	0.9500
C4—C5	1.4682 (11)	C16—H16	0.9500
C5—O1	1.2438 (10)	N3—H031	0.899 (16)
C11—C12	1.3949 (11)	N3—H032	0.893 (15)
C11—C16	1.3970 (12)	N4—N5	1.3128 (9)
C11—N5	1.4055 (10)	N5—H05	0.920 (14)
C12—C13	1.3916 (12)		
			
C5—N1—N2	113.92 (7)	C14—C13—C12	120.35 (9)
C5—N1—H01	127.2 (9)	C14—C13—H13	119.8
N2—N1—H01	118.6 (9)	C12—C13—H13	119.8
C3—N2—N1	105.54 (7)	C15—C14—C13	119.81 (8)
N2—C3—N3	124.13 (7)	C15—C14—H14	120.1
N2—C3—C4	111.24 (7)	C13—C14—H14	120.1
N3—C3—C4	124.56 (7)	C14—C15—C16	120.74 (9)
N4—C4—C3	124.84 (7)	C14—C15—H15	119.6
N4—C4—C5	129.43 (7)	C16—C15—H15	119.6
C3—C4—C5	105.40 (7)	C15—C16—C11	118.91 (8)
O1—C5—N1	127.60 (8)	C15—C16—H16	120.5
O1—C5—C4	128.47 (7)	C11—C16—H16	120.5
N1—C5—C4	103.89 (7)	C3—N3—H031	116.3 (10)
C12—C11—C16	120.73 (8)	C3—N3—H032	115.2 (9)
C12—C11—N5	117.65 (7)	H031—N3—H032	114.2 (14)
C16—C11—N5	121.62 (8)	C4—N4—N5	117.74 (7)
C13—C12—C11	119.44 (8)	N4—N5—C11	119.81 (7)
C13—C12—H12	120.3	N4—N5—H05	121.2 (9)
C11—C12—H12	120.3	C11—N5—H05	119.0 (9)
			
C5—N1—N2—C3	0.58 (10)	C16—C11—C12—C13	−0.78 (13)
N1—N2—C3—N3	−176.87 (8)	N5—C11—C12—C13	179.67 (7)
N1—N2—C3—C4	0.11 (9)	C11—C12—C13—C14	0.95 (13)
N2—C3—C4—N4	−174.62 (8)	C12—C13—C14—C15	−0.15 (14)
N3—C3—C4—N4	2.34 (13)	C13—C14—C15—C16	−0.83 (14)
N2—C3—C4—C5	−0.68 (9)	C14—C15—C16—C11	0.99 (14)
N3—C3—C4—C5	176.29 (8)	C12—C11—C16—C15	−0.18 (13)
N2—N1—C5—O1	176.90 (8)	N5—C11—C16—C15	179.36 (8)
N2—N1—C5—C4	−0.97 (9)	C3—C4—N4—N5	177.60 (7)
N4—C4—C5—O1	−3.32 (15)	C5—C4—N4—N5	5.17 (13)
C3—C4—C5—O1	−176.89 (8)	C4—N4—N5—C11	−175.90 (7)
N4—C4—C5—N1	174.52 (8)	C12—C11—N5—N4	173.50 (7)
C3—C4—C5—N1	0.96 (8)	C16—C11—N5—N4	−6.05 (12)

### Hydrogen-bond geometry (Å, º)

**Table:**

*D*—H···*A*	*D*—H	H···*A*	*D*···*A*	*D*—H···*A*
N5—H05···O1	0.920 (14)	2.164 (14)	2.8524 (9)	130.9 (11)
N5—H05···O1^i^	0.920 (14)	2.266 (14)	3.0176 (10)	138.6 (12)
N1—H01···N2^ii^	0.931 (14)	2.089 (14)	2.9272 (10)	149.0 (12)
N1—H01···N1^ii^	0.931 (14)	2.547 (14)	3.0692 (14)	115.8 (10)
N3—H031···N2^iii^	0.899 (16)	2.369 (16)	3.2424 (10)	163.9 (13)
N3—H032···O1^iv^	0.893 (15)	2.185 (15)	3.0428 (10)	161.0 (13)

Symmetry codes: (i) −*x*, −*y*+1, −*z*+1; (ii) −*x*, −*y*+1, −*z*+2; (iii) −*x*+1, −*y*+1, −*z*+2; (iv) *x*+1, *y*, *z*.
